# Supplementary material for: Netcombin: An algorithm for constructing optimal phylogenetic network from rooted triplets
Source: PLoS One. 2020 Sep 18;15(9):e0227842. doi: 10.1371/journal.pone.0227842 (PMC7500971; doi:10.1371/journal.pone.0227842)
Supplement: S4 File — (ZIP) [file pone.0227842.s004.zip › SequenceData-Triplet/sequenceData-10-20-30-0-Triplet/length 300, numseq 40, rate 10^-7/10 seq.pdf]

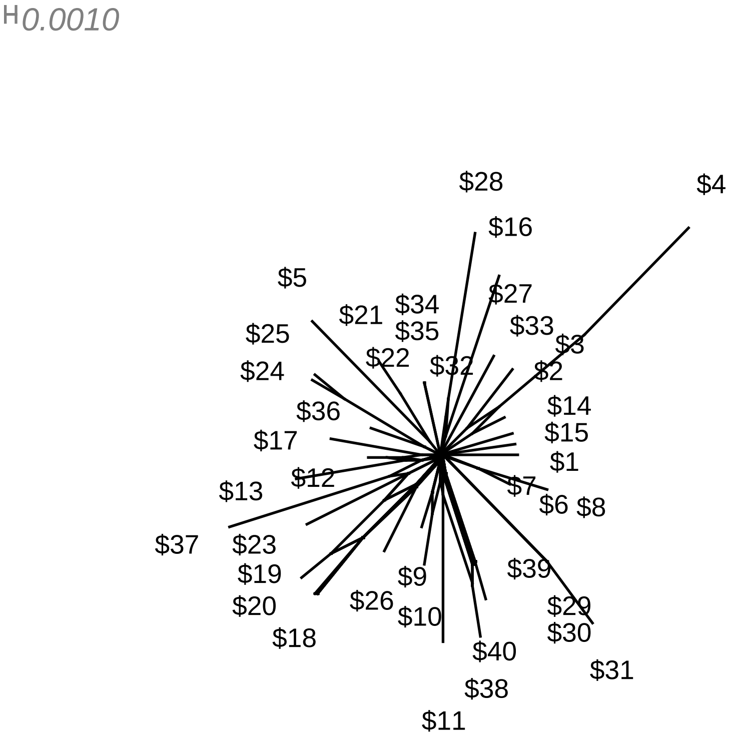
0.0010
$28
$4
$16
$5
$27
$34
$21
$33
$35
$25
$3
$22
$32
$2
$24
$14
$36
$15
$17
$1
$12
$7
$13
$6
$8
$37
$23
$39
$19
$9
$26
$29
$20
$10
$30
$18
$40
$31
$38
$11
